# Associations of childhood irritability and parenting profiles with youth suicide attempt: a longitudinal person-centered approach

**DOI:** 10.1017/S003329172510192X

**Published:** 2025-10-03

**Authors:** Cassandra Zephirin, Marie-Claude Geoffroy, Eszter Szekely, Léa C. Perret, Michel Boivin, Richard E. Tremblay, Sylvana M. Côté, Massimiliano Orri

**Affiliations:** 1 Department of Psychiatry, Faculty of Medicine and Health Sciences, McGill University, Montreal, QC, Canada; 2 McGill Group for Suicide Studies, Douglas Mental Health University Institute, Montreal, QC, Canada; 3 Lady Davis Institute for Medical Research, Jewish General Hospital, Montreal, QC, Canada; 4 Department of Epidemiology, Biostatistics, and Occupational Health, McGill University, Montreal, QC, Canada; 5 School of Psychology, Laval University, Quebec, QC, Canada; 6 Departments of Pediatrics and Psychology, University of Montreal, Montreal, QC, Canada; 7 Department of Social and Preventive Medicine, University of Montreal, Montreal, QC, Canada; 8 Danish Research Institute for Suicide Prevention, Mental Health Centre Copenhagen, Copenhagen, Denmark

**Keywords:** longitudinal, parenting, suicidal behavior, trajectory, youth irritability

## Abstract

**Background:**

Childhood irritability and harsh parenting are associated with youth suicide attempts. Parents’ harsh reactions have been associated with children’s irritable behavior. While studies have shown individual associations of irritability and parenting behaviors with suicide risk, few have considered these factors jointly. We aimed to identify profiles of children based on irritability and parenting during childhood and examine their associations with youth suicide attempt.

**Methods:**

Participants (*N* = 1626) were from the Québec Longitudinal Study of Child Development. Mothers reported on childhood irritability, harsh parenting, and positive parenting between ages 3.5 and 8; youth self-reported suicide attempt between ages 13 and 23.

**Results:**

We identified four profiles based on the joint development of irritability and parenting during childhood: (1) low irritability, low harsh parenting, and high positive parenting (30.3%); (2) moderate irritability, moderate harsh parenting, and high positive parenting (28.4%); (3) moderate irritability, moderate harsh parenting, and low positive parenting (26.6%); and (4) high irritability, high harsh parenting, and low positive parenting (14.8%). In logistic regression analyses, only children in the high irritability, high harsh parenting, and low positive parenting profile had higher odds of attempting suicide (OR = 2.51; 95% CI = 1.55–4.09) compared to those in the low irritability, low harsh parenting, and high positive parenting profile. This association remained significant (OR = 1.80; 95% CI = 1.03–3.15) in models adjusting for covariates.

**Conclusion:**

Children with chronically high irritability were also those experiencing the harshest parenting and the least positive parenting, as well as those most at risk of suicide attempt. Targeting both child and parental behavior may maximize suicide prevention efforts among children with high irritability.

## Introduction

Suicide attempt among youth is an important risk factor for suicide, a leading cause of death worldwide among 15- to 29-year-old youth (World Health Organization, 2023). Consequently, to prevent suicide in youth, it is important to identify modifiable risk factors for suicide attempt. Previous research has identified childhood irritability as a risk factor for adolescent suicide attempt (Forte et al., 2021; Galera et al., 2021; Orri et al., 2018; Orri et al., 2019). Irritability can be defined as a heightened proneness to anger that can reach impairing levels, and often happens in response to frustration (Leibenluft, 2017; Leibenluft et al., 2024; Leibenluft & Stoddard, 2013). Childhood irritability manifests itself clinically through the display of developmentally inappropriate and frequent temper outbursts that may take the form of verbal or behavioral expressions, such as aggression directed toward others, oneself, or objects (American Psychiatric Association, 2022). It is a symptom found in various mental disorders, both internalizing and externalizing ones (e.g. disruptive mood dysregulation disorder, oppositional defiant disorder, and major depressive disorder).

Several studies have shown that children with chronic or high levels of irritability are at increased risk for suicidal thoughts and behavior (Benarous et al., 2019; Leibenluft et al., 2023; Orri et al., 2019; Orri, Galera, et al., 2018; Orri, Perret, Turecki, & Geoffroy, 2018; Srinivasan et al., 2023). However, these studies have rarely accounted for contextual factors of the environment in which irritability manifests itself. Specifically, research has shown that children who are easily frustrated or have high levels of irritability evoke more negative reactions from their parents (Armour et al., 2018; Kiff, Lengua, & Zalewski, 2011; Lengua, 2006; Oliver, 2015). Parents’ harsh or hostile reactions have also been found to further increase children’s anger or irritability (Derella, Burke, Stepp, & Hipwell, 2020; Kiff et al., 2011; Oliver, 2015; Ravi et al., 2023). Some studies have also suggested that children’s irritable behavior does not increase when parents express responsive behaviors (Ravi et al., 2023; Van Den Bloom & Hoeksma, 1994). These studies fall in line with evidence that children’s characteristics and environmental factors, like parenting, engage in reciprocal transactions to drive development (Derella et al., 2020; Kiff et al., 2011; Oliver, 2015).

In several previous studies, harsh (e.g. hostile, strongly restrictive, and punitive) and positive (e.g. responsive, sensitive, and nurturing) parenting behaviors have also been identified as risk and protective factors, respectively, for suicidal ideation and behavior (Donath, Graessel, Baier, Bleich, & Hillemacher, 2014; Lai & McBride-Chang, 2001). However, studies that consider the joint influence of irritability and parenting behavior on suicide attempt are rare. To our knowledge, only one previous study using the Québec Longitudinal Study of Child Development (QLSCD) investigated whether harsh parenting practice in early adolescence mediated the association between childhood irritability and youth suicide attempt, finding no evidence for such an effect (Forte et al., 2021). In sum, while prior studies have investigated the role of irritability and parenting independently on suicidal behavior, research is scarce when it comes to studying these factors together in a systemic way. That is, groups at risk may be better identified by jointly looking at both individual characteristics (such as irritability traits) and characteristics of their environment (such as parental behavior), rather than studying how such characteristics co-vary across a whole sample. The former is known as a *person-centered approach*, as opposed to the latter, a *variable-centered approach* (Laursen & Hoff, 2006). The key difference is that a person-centered approach identifies groups at risk based on the joint consideration of both individual and parental characteristics and helps investigate whether these groups differ in terms of later suicide risk.

To guide our investigation, we drew on the diathesis–stress framework, which posits that individual vulnerabilities interact with environmental stressors to confer risk for psychopathology (Monroe & Simons, 1991). In this context, childhood irritability may represent a dispositional vulnerability that increases sensitivity to adverse caregiving environments, like harsh parenting. When such vulnerabilities (irritability) co-occur with negative experiences (harsh parenting), the likelihood of maladaptive outcomes, including suicide attempt, is heightened. This framework provides a useful lens for understanding how child-level and environmental factors may co-develop and jointly shape suicide risk across development.

Using data from the QLSCD, a 23-year birth cohort, the aim was to identify profiles of children based on both child irritability and parenting practices during childhood (3.5–8 years), and examine their longitudinal associations with suicide attempt by young adulthood (by 23 years). Characterizing children in the context of the family system and taking into account the behaviors of both children and parents would allow us to go beyond what has been done in previous literature, shedding light on the nuanced relationships among irritability, parenting, and suicidal behavior. This may help identify groups of children at higher risk for suicide attempt based on both child and parental behavior, providing valuable insights for tailoring interventions to address multiple elements of the family system.

## Methods

### Sample and participants

Participants were drawn from the QLSCD, a 23-year representative population-based birth cohort of children born in 1998/99 in Québec (Orri et al., 2021), conducted by the Institut de la Statistique du Québec. The original sample (N = 2120) was selected using a stratified random procedure based on living area and birth rate from the Québec Birth Registry. Mothers were included if gestation lasted 24–48 weeks and they spoke French or English. Children were followed from 5 months to 23 years of age (2021), with annual or biannual assessments during childhood and adolescence. Analyses were based on 1626 participants (~77% of the original sample) with available data on suicide attempt (ages 13, 15, 17, 20, or 23) and irritability and parenting (ages 3.5–8). Compared to the excluded sample, included participants had a higher proportion of females, higher socioeconomic status (SES), higher internalizing problems, lower harsh and positive parenting, and differed in family structure, but did not differ on other characteristics, including irritability (Supplementary Table S1). Ethical approval for the study was obtained from the Institut de la Statistique du Québec and the CHU Sainte-Justine Hospital Research Centre. Approval for the 2021 Special Round (age 23 data collection) was additionally obtained from the Douglas Research Center and CHU Sainte-Justine research ethics committees. Written informed consent was obtained from participants and/or parents at each data collection.

### Measures

**Childhood irritability.** The child’s most knowledgeable person (i.e. the mother in 98% of cases) reported on irritability between ages 3.5 and 8 years using items from the Social Behavior Questionnaire (Collet, Orri, Tremblay, Boivin, & Côté, 2023), which assesses a broad range of emotional and behavioral difficulties but was not designed specifically to measure irritability. Accordingly, items were selected to best capture this construct. Following prior work (Forte et al., 2021; Galera et al., 2021; Orri et al., 2019; Orri, Galera, et al., 2018), irritability was defined as temper tantrums and reactive aggression, measured with four items: three assessing *reactive aggression* (‘reacted in an aggressive manner when teased’, ‘when contradicted’, and ‘when something was taken away’) and one assessing *temper tantrums* (‘had temper tantrums/hot temper’). This definition aligns with both the behavioral/verbal anger manifestations and mood components outlined in the DSM-5 criteria for Disruptive Mood Dysregulation Disorder (American Psychiatric Association, 2022). Items were rated using a 3-point Likert scale (0 = never, 1 = sometimes, and 2 = often) based on behavior in the past 6 months. At each age (3.5, 4, 5, 6, and 8 years), irritability scores were calculated by averaging available items. As the temper tantrum was unavailable at ages 3.5, 4, and 5, these scores were based on the remaining three items (Supplementary Table S2). Age-specific irritability scores were used in profile analyses, and correlations between them are shown in Supplementary Table S3. Internal consistencies for each age were 0.70, 0.69, 0.78, 0.78, and 0.79, respectively. To empirically distinguish irritability from the hostile/defiant dimension of oppositional defiant disorder (ODD), we conducted a factor analysis using relevant ODD items (e.g. was defiant or refused to comply with adults’ requests or rules; did not seem to feel guilty after misbehaving; and punishment did not change their behavior) and irritability items. Results supported two distinct factors (Supplementary Table S4).

**Parenting behavior.** Parenting behaviors were reported by one parent at each wave; in 98% of cases, the respondent was the mother. Parents completed the Strayhorn and Weidman’s Parent Practices Scale, previously used in the Canadian National Longitudinal Survey of Children and Youth, when children were between 3.5 and 8 years of age. Two dimensions of parenting were examined: *harsh parenting*, assessed with 4–7 items measuring harsh/coercive behaviors such as giving punishments that depend on the parents’ mood, and using physical punishment (e.g. ‘how often did you hit him/her when difficult?’, ‘get angry when you punished him/her?’); and *positive parenting*, assessed with 5–10 items measuring responsive reactions to the child (e.g. ‘calmly discuss the problem’, ‘play sports, activities or games together’, and ‘praise the child’). Full item lists are available in Supplementary Tables S5 and S6. Parents rated the frequency of these behaviors over the past year on a 5-point Likert scale (e.g. 1–2 times/week to several times/day). Harsh and positive parenting scores were calculated at each age by averaging the respective items and used separately in subsequent analyses. Correlations between parenting scores across ages are shown in Supplementary Table S3. Internal consistencies for harsh parenting were 0.71, 0.67, 0.72, 0.72, and 0.64, and for positive parenting, 0.62, 0.63, 0.61, 0.60, and 0.59, respectively.

**Suicide attempt.** Suicide attempt was assessed at ages 13, 15, 17, 20, and 23 years. Participants were first asked a gate question: ‘In the past 12 months, did you ever seriously think of attempting suicide?’. Those who answered ‘yes’ were then asked, ‘How many times did you attempt suicide?’, with responses dichotomized as no or ≥ 1 attempt. At ages 20 and 23, participants were additionally asked whether they ‘ever went to the emergency department for a suicide attempt’ or ‘have ever been hospitalized for a suicide attempt’. A lifetime suicide attempt variable was derived based on a ‘yes’ response to any of these suicide attempt items. This composite approach was used to maximize case detection across adolescence and early adulthood.

**Adjustment variables.** We selected the following variables a priori to account for individual and family characteristics in adjusted models. Child sex (biological male or female) was extracted from birth medical records. Family SES, assessed between 1.5 and 8 years, was measured using an aggregate of 5 items relating to parental occupation, education, and annual gross income (range: −3 to 3, centered at 0; averaged across ages; Willms & Shields, 1996), with lower scores indicating lower SES. Maternal age at child’s birth, maternal depressive symptoms (mother reported using the Centre for Epidemiological Study Depression Scale), and family structure (intact, e.g. both biological parents; single parent; or blended, e.g. to partners with a child or children from previous relationships) were assessed at 5 months. To isolate the independent role of irritability, models were adjusted for externalizing problems (averaged hyperactivity–inattention and behavioral oppositional defiant disorder symptom scores) and internalizing problems, both assessed with the Social Behavior Questionnaire (parent report), where total scores were calculated by averaging scores at each age between 1.5 and 8 years.

### Statistical analysis

We used parallel process latent class growth analysis (i.e. multitrajectory modeling) in Mplus version 8.5 to jointly estimate developmental trajectories of childhood irritability, harsh parenting, and positive parenting from ages 3.5 to 8. This method enabled us to jointly model the longitudinal course of multiple variables (in our case, irritability, harsh parenting, and positive parenting), to identify distinct profiles characterized by the ways in which irritability and parenting develop together across childhood. We estimated models with one to five classes, each including intercept and slope parameters for all variables. Parameters were estimated using robust maximum likelihood, which handles missing data (full information maximum likelihood). Model selection was based on Bayesian information criterion (BIC; lower values indicate better fit), entropy (i.e. indicating the quality of the classification of the individuals in their most likely profile, ranging from 0 to 1, with values of ≥ 0.70 indicating good classification), and model interpretability. Once the best model was selected, participants were assigned to the profile with the highest posterior probability of class membership.

Logistic regressions in R estimated odds ratios (ORs) for suicide attempt across identified profiles (in comparison to a reference profile), using unadjusted models, models adjusted for child sex and SES, and fully adjusted models including all covariates. We also tested profile-by-sex interactions. Missing data on the covariates were imputed using multiple imputations (*K* = 20) to avoid loss of participants in the multivariable models. Furthermore, inverse probability weighting, in which weights represent the probability to be included in the sample estimated from baseline sex, SES, and internalizing problems (i.e. the baseline variables independently associated with attrition), was used in our models to compensate for bias arising from differential attrition between participants included and non-included in the analysis.

## Results

The study included 1626 children who were followed up to 23 years of age; 780 (48%) were boys and 846 (52%) were girls. Sociodemographic characteristics of the sample are reported in Table 1. Correlations between irritability and parenting are reported in Supplementary Table S3, and mean scores of irritability and parenting variables are reported in Supplementary Table S7.

**Table 1.** Descriptive statistics of the included sample stratified by sex^a^

	Included sample	Males	Females	*p*
*n*	1626	780 (48%)	846 (52%)	
**Child characteristics**				
Low birthweight (< 2500), *n* (%)	50 (3.1%)	25 (3.2%)	25 (3.0%)	.886
Birth order, *n* (%)				.546
1	719 (44.2%)	343 (44.0%)	376 (44.4%)	
2	651 (40.0%)	321 (41.2%)	330 (39.0%)	
3+	256 (15.7%)	116 (14.9%)	140 (16.5%)	
Externalizing problems, mean (*SD*)	2.20 (0.85)	2.39 (0.86)	2.09 (0.82)	<.001**
Internalizing problems, mean (*SD*)	1.73 (0.96)	1.73 (0.98)	1.73 (0.95)	.893
**Child outcomes**				
Irritability, mean (*SD*)	0.70 (0.41)	0.73 (0.40)	0.67 (0.41)	.003**
Suicide attempt, *n* (%)	145 (8.9%)	49 (6.3%)	96 (11.3%)	<.001**
**Family characteristics**				
Positive parenting, mean (*SD*)	6.18 (0.88)	6.19 (0.91)	6.16 (0.85)	.438
Harsh parenting, mean (*SD*)	2.76 (0.97)	2.86 (1.00)	2.66 (0.93)	<.001**
Socioeconomic status (SES), mean (*SD*)	0.02 (0.95)	0.01 (0.92)	0.03 (0.98)	.724
Family structure, *n* (%)				.588
Intact	1324 (81.7%)	636 (81.7%)	688 (81.7%)	
Single	114 (7.0%)	59 (7.6%)	55 (6.5%)	
Blended	182 (11.2%)	83 (10.7%)	99 (11.8%)	
Maternal depression, mean (*SD*)	1.37 (1.32)	1.41 (1.37)	1.34 (1.27)	.233
Paternal depression, mean (*SD*)	0.99 (0.94)	1.01 (0.94)	0.97 (0.94)	.442
Maternal age, mean (*SD*)	29.39 (5.16)	29.27 (5.21)	29.51 (5.12)	.354
Paternal age, mean (*SD*)	32.26 (5.52)	32.44 (5.64)	32.09 (5.40)	.222

Note: *p < .05. **p < .01.

a Data were compiled from the final master file of the Québec Longitudinal Study of Child Development (1998–2021), Gouvernement du Québec, and l’Institut de la Statistique du Québec. Externalizing, internalizing problems, and SES, measured from 1.5 to 8 years; irritability, positive, and harsh parenting measured from 3.5 to 8 years. Family structure, maternal age and depression, and paternal age and depression measured at 5 months.

### Profiles of irritability and parenting behaviors

**Figure 1.**
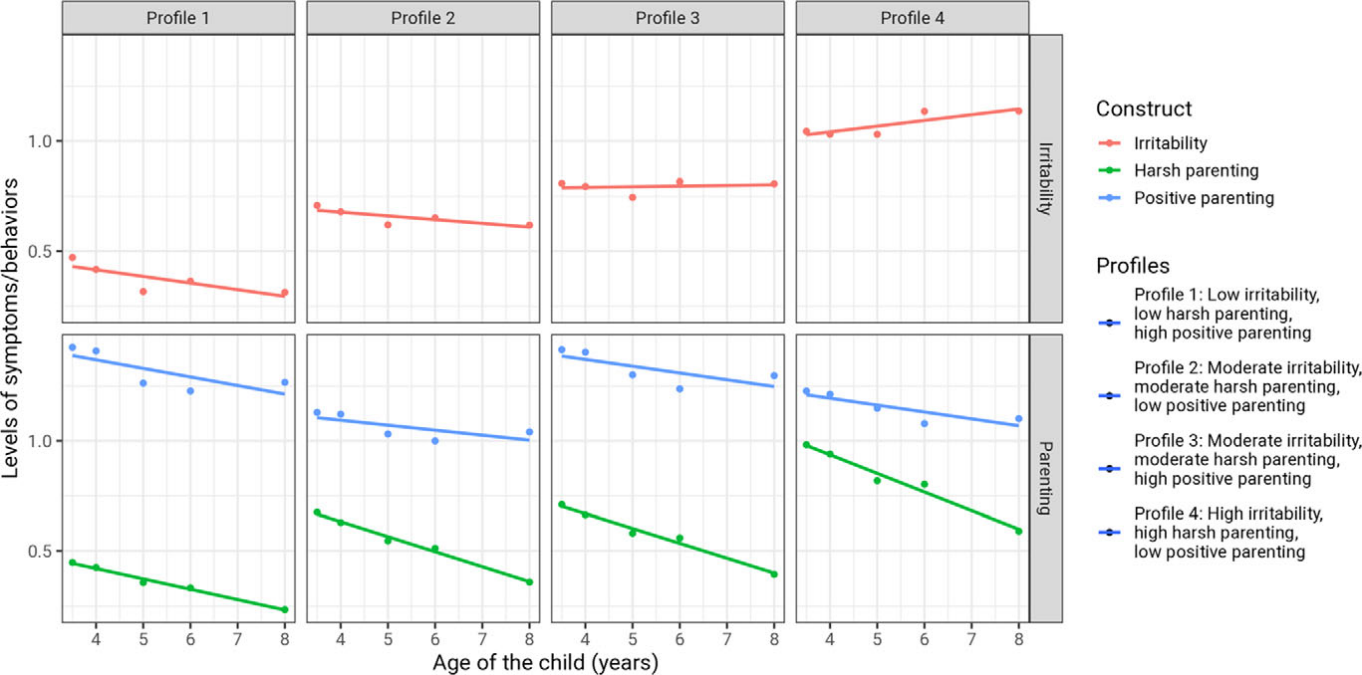
Multitrajectories of childhood irritability and harsh/positive parenting. The figure shows the mean level of irritability, harsh parenting, and positive parenting (y-axis) across the observation period (x-axis) by profile (panels and colors). An alternative representation of our multitrajectories is provided in Supplementary Figure S1. Data were compiled from the final master file of the Québec Longitudinal Study of Child Development (1998–2021), Gouvernement du Québec, and l’Institut de la Statistique du Québec.

The best model identified the following four profiles (Figure 1; see also Supplementary Tables S7, S8, and S9): (1) low irritability, low harsh parenting, and high positive parenting (492 [30.3%]); (2) moderate irritability, moderate harsh parenting, and high positive parenting (461 [28.4%]); (3) moderate irritability, moderate harsh parenting, and low positive parenting (432 [26.6%]); and (4) high irritability, high harsh parenting, and low positive parenting (241 [14.8%]). Sociodemographic characteristics stratified by trajectories are available in Supplementary Table S10. Main group differences include child sex, birth order, internalizing and externalizing problems, SES, family structure, maternal and paternal depression, and maternal age. Although the five-class model yielded slightly lower BIC and AIC values, the additional class did not represent a qualitatively distinct pattern (Supplementary Table S8). Instead, it appeared to divide one of the four-class groups into two smaller, conceptually overlapping subgroups, which reduced interpretability and parsimony. The four-class solution offered a better balance between statistical fit and theoretical clarity, identifying meaningful subgroups varying in both irritability and parenting trajectories. Entropy for the four-class solution was 0.767, indicating good classification.

### Associations of childhood irritability and parenting profiles with adolescent suicide attempt

The proportion of adolescents presenting with a suicide attempt in each profile is shown in Table 2. Suicide attempt was reported by 145 participants (8.9% of our sample). Overall, females had higher rates of suicide attempt relative to males. Of the children in the high irritability, high harsh parenting, and low positive parenting profile, 35 of 241 attempted suicide (14.5%). Forty-two of 461 (9.1%) children in the moderate irritability, moderate harsh parenting, and high positive parenting profile, and 33 of 432 (7.6%) children in the moderate irritability, moderate harsh parenting, and low positive parenting profile, attempted suicide. Lastly, of the children in the low irritability, low harsh parenting, and high positive parenting profile, 35 of 492 (7.1%) attempted suicide. Among participants who reported a suicide attempt based on past year assessments (*n* = 122), the average age at first reported attempt was 16.7 years (range: 13–23 years). The majority (*n* = 99; 81.1%) reported a single attempt, while 23 (18.9%) participants reported repeated suicide attempts. An additional 23 individuals who attempted suicide were identified based solely on the hospitalization questions at ages 20 and 23, yielding a final count of 145 individuals with a lifetime suicide attempt. Full descriptive statistics are provided in Supplementary Table S11.

**Table 2.** Suicide attempt rates in youth by childhood irritability and parenting trajectory^a^

	Suicide Attempt	
Profile	*n*	%
Low irritability, low harsh parenting, and high positive parenting (*n* = 492, 30.3%)	35	7.1
Moderate irritability, moderate harsh parenting, and low positive parenting (*n* = 432, 26.6%)	33	7.6
Moderate irritability, moderate harsh parenting, and high positive parenting (*n* = 461, 28.4%)	42	9.1
High irritability, high harsh parenting, and low positive parenting (*n* = 241, 14.8%)	35	14.5
Whole sample (*N* = 1626)	145	8.9

a Data were compiled from the final master file of the Québec Longitudinal Study of Child Development (1998–2021), Gouvernement du Québec, and l’Institut de la Statistique du Québec.

Table 3 shows ORs and 95% CIs for all profiles compared with the low irritability, low harsh parenting, and high positive parenting profile (reference). We found that children with chronically high irritability, high harsh parenting, and low positive parenting had two and a half times greater odds of attempting suicide (OR, 2.51; 95% CI, 1.55–4.09) compared to those in the reference profile; these odds remained similar after adjusting for child sex and SES (OR, 2.36; 95% CI, 1.43–3.88). In models adjusting for child sex, SES, family structure, maternal age, and maternal depression, children with high irritability, high harsh parenting, and low positive parenting continued to demonstrate a significantly higher risk for attempting suicide in adolescence (OR, 2.28; 95% CI, 1.37–3.78). In fully adjusted models, children with high irritability, high harsh parenting, and low positive parenting still demonstrated a significantly greater risk for attempting suicide in adolescence (OR, 1.80; 95% CI, 1.03–3.15). Children in the moderate irritability, moderate harsh parenting, and high positive parenting profile (adjusted OR, 1.21, 95% CI, 0.74–1.98) and the moderate irritability, moderate harsh parenting, and low positive parenting profile (adjusted OR, 1.01, 95% CI, 0.61–1.69) exhibited odds of attempting suicide comparable to those in the reference group; in other words, they did not significantly differ from the reference group in their odds of suicide attempts. Results were consistent with non-imputed data (Supplementary Table S12). To provide a more comprehensive understanding of group differences, we conducted additional analyses comparing the high-risk profile with all other profiles. Results indicated that the group characterized by high irritability, high harsh parenting, and low positive parenting exhibited significantly higher odds of suicide attempts compared to all other profiles, emphasizing the elevated risk for children with this specific combination of factors (see Supplementary Tables S13 and S14).

**Table 3.** Associations of childhood profiles of irritability and parenting at 3.5–8 years of age with youth suicide attempt, imputed data^a^

	Suicide Attempt	Outcome, OR (95% CI)		
Profile	Unadjusted	Adjusted^b^	Adjusted^c^	Adjusted^d^
Low irritability, low harsh parenting, and high positive parenting	1 [Reference]	1 [Reference]	1 [Reference]	1 [Reference]
Moderate irritability, moderate harsh parenting, and low positive parenting	1.15 (0.70–1.87)	1.12 (0.68–1.84)	1.12 (0.68–1.85)	1.01 (0.61–1.69)
Moderate irritability, moderate harsh parenting, and high positive parenting	1.46 (0.92–2.32)	1.41 (0.88–2.25)	1.41 (0.88–2.25)	1.21 (0.74–1.98)
High irritability, high harsh parenting, and low positive parenting	2.51 (1.55–4.09)	2.36 (1.43–3.88)	2.28 (1.37–3.78)	1.80 (1.03–3.15)

Abbreviation: OR, odds ratio.

a Data were compiled from the final master file of the Québec Longitudinal Study of Child Development (1998–2021), Gouvernement du Québec, and l’Institut de la Statistique du Québec.

b Adjusted for child sex and socioeconomic status (SES).

c Adjusted for child sex, SES, family structure, maternal age, and maternal depression.

d Adjusted for child sex, SES, family structure, maternal age, maternal depression, child internalizing, and externalizing problems.

Interactions with sex were examined to investigate whether associations differed for males and females, but none were statistically significant; therefore, stratified analyses were not conducted.

## Discussion

This prospective longitudinal population-based study used a person-centered approach to delineate distinct developmental profiles of children by examining both childhood irritability and parenting behaviors measured repeatedly between 3.5 and 8 years. Furthermore, we investigated how the identified profiles were associated with later youth suicide attempt, and found that the co-occurrence of irritability and parenting behavior characterized by frequent harsh and infrequent positive behaviors was associated with elevated risk for youth suicide attempt.

### Profiles of irritability and parenting behaviors

To our knowledge, this is the first study that jointly modeled childhood irritability and parenting behaviors to identify distinct subgroups of children. The best model identified four profiles. In all these profiles, we observed that the levels of irritability and parenting were persistent throughout childhood, such that, children with the highest levels of irritability, harsh parenting, and lowest levels of positive parenting at 3.5 years of age maintained these characteristics at 8 years of age (i.e. they conserved rank stability although the overall frequency of some behavior changed over time in a similar way for all groups). This may suggest that there is little variation in parent–child interpersonal behaviors, possibly resulting in repetition of the same behavioral patterns for long periods of time. Specifically, we observed that children displaying chronically high levels of irritability also experienced high levels of harsh parenting and low levels of positive parenting over the course of early and middle childhood. Conversely, children displaying low levels of irritability also had parents who consistently showed positive, non-harsh parenting behaviors. Interestingly, no profile emerged in which highly irritable children were exposed to non-harsh and positive parenting, possibly suggesting that parenting practices and irritability in children are not mutually exclusive and are indeed difficult to tease apart. These results show the utility of a person-centered approach for considering multiple aspects (here, child behavior and parent behavior) rather than studying each aspect in isolation.

Overall, our findings align with research using variable-centered approaches. Such research suggests that children characterized by irritability or other forms of negative emotionality typically tend to elicit negative responses from their parents (Armour et al., 2018; Kiff et al., 2011; Lengua, 2006; Oliver, 2015), and when parents react negatively (e.g. harshly or with hostility) to their children’s irritable behavior, it may exacerbate the child’s irritability into adolescence (Derella et al., 2020; Kiff et al., 2011; Oliver, 2015; Ravi et al., 2023). Prior research suggests that irritability may influence different aspects of negative parenting behavior. For example, Lengua and colleagues (2005) investigated this using multiple regression analyses and found that child irritability predicted greater parental inconsistency (a form of negative parental control), and parental inconsistent discipline predicted greater fearfulness and irritability in children. These findings are in line with the idea that reciprocal associations between temperament and parenting exist, such that inconsistent discipline in parents may exacerbate negative emotionality in children, while irritability in children may evoke inconsistent behavior from parents (Lengua & Kovacs, 2005). Furthermore, Lengua (2006) also demonstrated that higher initial parental rejection predicted increases in child fear and irritability, and higher initial irritability in children predicted increases in parental inconsistency. Overall, these findings suggest the possibility that child temperament and parenting may mutually influence changes in one another, and together, they may influence child outcomes into adolescence (Lengua, 2006). Future studies are needed to better understand the reciprocal associations between child irritability and parenting behavior, as our person-centered approach precludes a fine understanding of the parent–child interaction at play. For example, definitive inference regarding directionality – whether the child’s reaction is a response to parental behavior or vice versa – would require a different, variable-centered approach, complementary to the one used in our study. Thus, while our findings are valuable for identifying at-risk groups, they do not explicitly address the dynamic interplay between these variables, as this was beyond the scope of our analysis. Understanding this remains a critical area for future research, which could provide more comprehensive insights into how irritability and parenting influence one another over time.

### Associations of childhood irritability and parenting profiles with adolescent suicide attempt

We found that one in seven children (14.5%) with high levels of irritability, harsh parenting, and low levels of positive parenting attempted suicide by age 23, compared to 1 in 14 children (7.1%) attempting suicide among those with low levels of irritability, harsh parenting, and high levels of positive parenting. This increased risk was not fully explained by socioeconomic factors and concurrent psychopathology considered in our multivariable models. Importantly, we found that children in this profile were at increased risk of suicide attempt compared to all other profiles, further highlighting the specificity of this combination of individual- and family-level characteristics. Previous studies have demonstrated that irritability (Forte et al., 2021; Orri et al., 2019; Orri, Galera, et al., 2018) and harsh parenting (Donath et al., 2014; Kingsbury, Sucha, Manion, Gilman, & Colman, 2020a; Lai & McBride-Chang, 2001) in childhood are significant risk factors for suicide-related outcomes (i.e. suicidal ideation and suicide attempt) in youth. Our findings align well with data showing that parents of children who engage in suicidal behaviors display less parental warmth (Lai & McBride-Chang, 2001) and engage in more rejecting–neglecting ways (Donath et al., 2014). However, while previous studies have explored irritability and parenting separately, our findings uniquely contribute to the literature by jointly investigating these factors. Specifically, our study identifies subgroups of individuals characterized by the simultaneous presence of irritability and specific parenting behaviors throughout childhood, and highlights the heightened risk of suicide attempts in those exposed to a specific combination of these factors.

A prior study conducted on the QLSCD tested whether harsh parenting in early adolescence (age 13 years) mediated the association between childhood irritability and youth suicide attempt, but found no evidence supporting such an effect (Forte et al., 2021). This difference in findings may indicate that what increases suicide attempt risk may be the cumulative presence of irritability and harsh/non-positive parenting, rather than a mechanism in which irritability increases harsh parenting, in turn increasing suicide attempt risk. However, it is also important to note that this study solely assessed harsh parenting at 13 years of age. Given that irritability and parenting may operate differently across various developmental stages (i.e. childhood vs. adolescence) (Lengua & Kovacs, 2005), it is possible that the findings from the two studies are not directly comparable. Future studies measuring irritability and parenting into adolescence may clarify whether our findings are the same when different developmental periods are considered.

Concerning the role of positive parenting, the literature has reported mixed findings. On the one hand, a number of studies have identified positive parenting as a protective factor for later child behavior problems (i.e. externalizing and affective problems), including suicide-related outcomes (Chronis et al., 2007; Eisenberg et al., 2005; Ezpeleta, Penelo, De La Osa, Navarro, & Trepat, 2019; Lai & McBride-Chang, 2001; Mackin, Perlman, Davila, Kotov, & Klein, 2017; McKee et al., 2007). On the other hand, other studies did not observe a protective association of positive parenting on subsequent behavioral problems in childhood or suicide-related outcomes (Kingsbury et al., 2020a; Kingsbury, Sucha, Manion, Gilman, & Colman, 2020b; Petitclerc, Boivin, Dionne, Zoccolillo, & Tremblay, 2009). In line with the latter, our findings did not support such a protective association. For example, children presenting with moderate irritability, harsh parenting, and low positive parenting showed a similar risk of later suicide attempt as those with moderate irritability, harsh parenting, and high positive parenting. Should positive parenting have acted as a buffer, we would have expected to observe a lower risk of attempting suicide in the group with moderate irritability, harsh parenting, and high positive parenting, given that they only differed in their levels of positive parenting from the group with moderate irritability, harsh parenting, and low positive parenting. Rather, our findings suggest that it may be the combination of irritability and harsh parenting, rather than the influence of positive parenting, that may play a more critical role in determining the risk of suicide attempt. Contrary to our expectations, suicide attempt risk among youth in the moderate irritability, high harsh parenting, and high positive parenting profile was comparable to that of those in the moderate irritability, high harsh parenting, and low positive parenting profile. While this may suggest that the presence of high positive parenting did not mitigate risk in the context of harsh parenting, this interpretation should be made cautiously. The positive parenting measure demonstrated only modest internal consistency (α = 0.59–0.63), which may have limited our ability to detect buffering effects. However, this level of reliability is consistent with prior use of the scale in the QLSCD cohort and has shown predictive utility in previous research (Boyle et al., 2004; La Buissonnière-Ariza et al., 2019; Laurin, Joussemet, Tremblay, & Boivin, 2015). It is also possible that the co-occurrence of warmth and harshness reflects inconsistent or authoritarian parenting styles, which may not confer the same protective effects/benefits as consistently positive parenting approaches. The diathesis–stress model provides a useful theoretical framework for understanding the potential contribution of irritability and harsh parenting in the development of suicide risk (Arafat, Menon, Dinesh, & Kabir, 2022; Perquier et al., 2021; Ravi et al., 2023; Zuckerman, 1999). Childhood irritability can be considered an individual vulnerability factor that not only subjects children to a dispositional proneness to anger but also makes children increasingly susceptible and sensitive to their environment. Exposure to early rearing environments that are highly negative and are often characterized by high levels of coercive and inconsistent parenting, as is the case for irritable children (Kiff et al., 2011; Lengua & Kovacs, 2005), may result in poorer outcomes in adolescence, potentially exacerbating the risk of suicide in irritable youth. In addition, investigations of irritability have identified a dysfunction in the threat processing of irritable children (Brotman, Kircanski, Stringaris, Pine, & Leibenluft, 2017). Children with severe irritability typically direct more attention toward threatening, angry faces than to neutral faces (Hommer et al., 2014), interpret ambiguous faces as angry (Stoddard et al., 2016), and demonstrate differences in their processing of happy faces compared to children with low levels of irritability (Tseng et al., 2016). Taken together, this literature suggests that irritable children may be hypersensitive to harsh reactions from parents and hyposensitive to positive responses from parents (Ravi et al., 2023). Further investigation is necessary to clarify the distinct mechanisms at play between irritability and different parenting behaviors.

### Implications of the findings for clinical practice

Our findings underscore that addressing suicide risk in irritable children may require a comprehensive and holistic approach to intervention, acknowledging the interplay between childhood irritability and parenting behaviors. Given that children at the highest risk for suicide attempt were those presenting with chronically high irritability symptoms and exposed to parenting behaviors characterized by harshness and low positivity, a systemic approach targeting both aspects may be necessary for effective prevention efforts. For example, child-focused interventions fostering emotional regulation in children, paired with parenting interventions that reduce harsh parenting, may maximize the prevention of long-term risk of suicide attempt. Parent–Child Interaction Therapy (PCIT) is one such evidence-based program that has demonstrated efficacy in improving parenting practices and reducing child externalizing behaviors, with additional benefits for child emotion regulation (Thomas, Abell, Webb, Avdagic, & Zimmer-Gembeck, 2017). These findings advocate for an approach that takes into account the family context for suicide prevention among irritable children (Frey & Hunt, 2018).

### Strengths and limitations

This study utilized a large population representative birth cohort of children followed from 5 months to 23 years of age, with prospective behavioral assessments of irritability and parenting behaviors at five different time points throughout childhood, and self-reports of suicide attempt across five waves between ages 13 and 23. It relies on an innovative multivariable longitudinal person-centered approach to identify clinically relevant subgroups of children. Despite these strengths, limitations should also be acknowledged. First, due to sample attrition, we conducted analyses on 77% of the original sample. Although the included and excluded samples were broadly comparable demographically, and weights were applied to compensate for bias due to differential attrition, caution should be exercised when generalizing our findings to the larger Québec population. Notably, participants from lower SES backgrounds and males were underrepresented in the analytic sample relative to the original cohort. This selective retention may have led to conservative estimates of suicide attempt prevalence and potentially underestimated the strength of associations between childhood profiles and suicide risk. A second limitation of the study is its observational design, which limits the ability to establish causal relationships between variables. Nevertheless, it offers valuable insights into associations that warrant further experimental investigation. Third, although we adjusted for a range of sociodemographic covariates, we could not account for potential shared genetic or familial liability. Through passive gene–environment correlation, parents may transmit heritable risk for irritability while also providing less supportive caregiving environments. Evocative processes may also be at play, as irritable children may elicit harsher responses from parents, reinforcing maladaptive parent–child dynamics over time (Kingsbury et al., 2020b, 2020a). These possibilities suggest that the observed associations may be partially confounded by unmeasured heritable influences on both child temperament and parenting behaviors, and thus should be interpreted with appropriate caution. Fourth, although our models accounted for several important sociodemographic and psychosocial variables (e.g. maternal depression, SES, family structure, and child behavioral symptoms), residual confounding remains a possibility. Unmeasured factors, such as exposure to adverse life events, may also contribute to suicide risk and potentially confound associations between childhood profiles and later outcomes. Accordingly, these findings should be interpreted with caution, and causality cannot be inferred. Fifth, similar to most large longitudinal population-based studies, the instruments used to measure childhood symptoms are not clinical tools designed to evaluate clinical conditions, but rather to evaluate a spectrum of behaviors and emotional dimensions. In particular, it is important to consider potential variations in how the irritability construct was measured when comparing our findings with other studies, since our items were not taken from a scale specifically designed to measure childhood irritability. To that same end, parenting items across ages may exhibit some variability; however, this variation is reflective of developmentally appropriate questions. Sixth, not all items used to measure irritability were available at all time points. Despite the high correlation among the items, the unavailability of items may have influenced the measured construct. Seventh, self-report measures are susceptible to several limitations and biases inherent in their nature, including social desirability, particularly when it comes to reporting on harsh behaviors in the parent–child relationship. For instance, parents may be less inclined to report on their own harsh behaviors while potentially overreporting their positive parenting behaviors. Therefore, this may have led to some underreporting of harsh behaviors. Eighth, the internal consistency of the positive parenting measure was modest across time points (α = 0.59–0.63), which may reduce confidence in the construct’s reliability and attenuate its associations with downstream outcomes. As such, null effects involving this variable should be interpreted with caution. Ninth, due to the relatively low prevalence of suicide attempts, we considered a measure of any suicide attempt throughout adolescence and young adulthood as our outcome. This strategy aimed to maximize statistical power but may have introduced some heterogeneity. Indeed, aggregating suicide attempts across such a broad developmental span (ages 13–23) may also obscure distinctions based on age of onset of suicide attempt (e.g. between early vs. late-onset), or between single and repeated attempts (Geoffroy et al., 2022; Geoffroy, Orri, Girard, Perret, & Turecki, 2021). Future studies with larger samples and time-to-event data will be essential to clarify these developmental trajectories. Furthermore, since the past year, suicide attempts have been evaluated every other year; suicide attempts occurring between waves may have gone unreported. Our use of lifetime hospitalization questions at ages 20 and 23 helped identify additional cases that may have been missed by past-year recall, partially addressing the underreporting. Tenth, the use of parent-report measures for both irritability and parenting may have introduced bias related to shared-method variance, potentially influencing the results. Parents’ own emotion regulation difficulties may also have influenced their reporting, affecting how they describe both their own parenting behaviors, as well as their child’s irritability. Finally, parenting behaviors were reported by a single informant, predominantly mothers (98% of cases). While this offers consistency in reporting across waves, it may limit insight into potential variation in parenting practices between caregivers. Similarly, only maternal characteristics were included as covariates, which may restrict our understanding of broader family-level influences. Future studies incorporating multi-informant perspectives and paternal variables may offer a more comprehensive understanding of family influences/parent–child dynamics.

## Conclusions

By investigating the co-development of irritability and parenting behavior across childhood, our study identified a group of children characterized by chronically high irritability who also experienced high levels of harsh parenting and low levels of positive parenting. These children were at increased risk of suicide attempt during adolescence and young adulthood. The difficulties in dissociating child behavioral/mood aspects from parenting behaviors suggest that interventions combining both behavioral interventions for children and parenting interventions may maximize the changes to reduce later suicide risk among irritable children.

## Supporting information

Zephirin et al. supplementary material Zephirin et al. supplementary material

## Supplementary material

The supplementary material for this article can be found at http://doi.org/10.1017/S003329172510192X.
