# Supplementary material for: Associations of childhood irritability and parenting profiles with youth suicide attempt: a longitudinal person-centered approach
Source: Psychol Med. 2025 Oct 3;55:e292. doi: 10.1017/S003329172510192X (PMC12527484; doi:10.1017/S003329172510192X)
Supplement: Zephirin et al. supplementary material [file S003329172510192Xsup001.docx]

**Supplementary material**

**Associations of childhood irritability and parenting profiles with youth suicide attempt:**

**A longitudinal person-centered approach**

Cassandra Zephirin, Marie-Claude Geoffroy, Eszter Szekely, Léa C. Perret, Michel Boivin, Richard E. Tremblay, Sylvana M. Côté, Massimiliano Orri

**Table of content**

**Table S1.** Descriptive statistics of included sample vs. excluded sample

**Table S2**. Irritability items at each age

**Table S3**. Correlations between irritability and parenting

**Table S4.** Factor analysis

**Table S5**. Harsh parenting items at each age

**Table S6**. Positive parenting items at each age

**Table S7**. Mean scores of irritability and parenting variables for the overall sample and by profile

**Table S8**. Model fit indices

**Table S9.** Parameters estimates from the 4-latent class growth model of irritability and parenting

**Table S10.** Descriptive statistics stratified by trajectories of childhood irritability and parenting

**Table S11**. Descriptive statistics of suicide attempt at each age

**Table S12**. Associations of childhood profiles of irritability and parenting at 3.5 to 8 years of age with youth suicide attempt, non-imputed data

**Table S13**. Associations of childhood profiles of irritability and parenting at 3.5 to 8 years of age with youth suicide attempt based on different reference categories, non-imputed data

**Table S14**. Associations of childhood profiles of irritability and parenting at 3.5 to 8 years of age with youth suicide attempt based on different reference categories, imputed data

**Figure S1.** Profiles of irritability, harsh parenting, and positive parenting (alternative representation)

**Table S1**. Descriptive statistics of included sample vs. excluded sample.^a^

|  | **Included sample** | **Excluded sample** | ***p*** |
| --- | --- | --- | --- |
| *n* | 1626 | 494 |  |
| **Child characteristics** |  |  |  |
| Child sex = girls, *n* (%) | 846 (52.0%) | 194 (39.3%) | <.001** |
| Low birthweight (<2500), *n* (%) | 50 (3.1%) | 19 (3.8%) | .485 |
| Birth order, *n* (%) |  |  | .588 |
| 1 | 719 (44.2%) | 217 (43.9%) |  |
| 2 | 651 (40.0%) | 190 (38.5%) |  |
| 3+ | 256 (15.7%) | 87 (17.6%) |  |
| Externalizing problems, mean (*SD*) | 2.20 (0.85) | 2.26 (1.01) | .211 |
| Internalizing problems, mean (*SD*) | 1.73 (0.96) | 1.45 (1.07) | <.001** |
| Irritability, mean (*SD*) | 0.70 (0.41) | 0.72 (0.45) | .570 |
| **Family characteristics** |  |  |  |
| Positive parenting, mean (*SD*) | 6.18 (0.88) | 6.41 (0.99) | <.001** |
| Harsh parenting, mean (*SD*) | 2.76 (0.97) | 2.96 (1.14) | <.001** |
| Socioeconomic status (SES), mean (*SD*) | 0.02 (0.95) | -0.27 (1.01) | <.001** |
| Family structure, *n* (%) |  |  | .005* |
| Intact | 1324 (81.7%) | 382 (77.6%) |  |
| Single | 114 (7.0%) | 57 (11.6%) |  |
| Blended | 182 (11.2 %) | 53 (10.8%) |  |
| Maternal depression, mean (*SD*) | 1.37 (1.32) | 1.50 (1.41) | .065 |
| Paternal depression, mean (*SD*) | 0.99 (0.94) | 1.03 (1.03) | .493 |
| Maternal age, mean (*SD*) | 29.39 (5.16) | 28.99 (5.43) | .134 |
| Paternal age, mean (*SD*) | 32.26 (5.52) | 32.26 (6.05) | .996 |

*Note.* ^a^Data were compiled from the final master file of the Québec Longitudinal Study of Child Development (1998-2021), Gouvernement du Québec, and l’Institut de la Statistique du Québec. Externalizing, internalizing problems, and SES, measured from 1.5 to 8y; irritability, positive, and harsh parenting measured from 3.5y to 8y. Family structure, maternal age and depression, and paternal age and depression, measured at 5 months.

**p* < .05. ***p* < .01.

**Table S2**. Irritability items at each age.

| **Irritability ages** | **Items** |
| --- | --- |
| 3.5y, 4y, 5y, 6y, 8y | Reacted in an aggressive manner when contradicted |
| 3.5y, 4y, 5y, 6y, 8y | Reacted in an aggressive manner when teased |
| 3.5y, 4y, 5y, 6y, 8y | Reacted in an aggressive manner when something was taken away from him/her |
| 6y, 8y | Had temper tantrums/hot temper |

**Table S3**. Correlations between irritability and parenting.^a^

|  | **1** | **2** | **3** | **4** | **5** | **6** | **7** | **8** | **9** | **10** | **11** | **12** | **13** | **14** | **15** |
| --- | --- | --- | --- | --- | --- | --- | --- | --- | --- | --- | --- | --- | --- | --- | --- |
| 1 Irritability 3.5y | 1.00 |  |  |  |  |  |  |  |  |  |  |  |  |  |  |
| 2 Irritability 4y | 0.49 | 1.00 |  |  |  |  |  |  |  |  |  |  |  |  |  |
| 3 Irritability 5y | 0.43 | 0.48 | 1.00 |  |  |  |  |  |  |  |  |  |  |  |  |
| 4 Irritability 6y | 0.43 | 0.43 | 0.49 | 1.00 |  |  |  |  |  |  |  |  |  |  |  |
| 5 Irritability 8y | 0.40 | 0.45 | 0.49 | 0.60 | 1.00 |  |  |  |  |  |  |  |  |  |  |
| 6 Harsh parenting 3.5y | 0.30 | 0.28 | 0.30 | 0.30 | 0.29 | 1.00 |  |  |  |  |  |  |  |  |  |
| 7 Harsh parenting 4y | 0.21 | 0.32 | 0.27 | 0.28 | 0.31 | 0.64 | 1.00 |  |  |  |  |  |  |  |  |
| 8 Harsh parenting 5y | 0.23 | 0.28 | 0.39 | 0.35 | 0.35 | 0.57 | 0.61 | 1.00 |  |  |  |  |  |  |  |
| 9 Harsh parenting 6y | 0.24 | 0.29 | 0.32 | 0.45 | 0.40 | 0.54 | 0.58 | 0.63 | 1.00 |  |  |  |  |  |  |
| 10 Harsh parenting 8y | 0.20 | 0.22 | 0.26 | 0.26 | 0.38 | 0.43 | 0.47 | 0.49 | 0.53 | 1.00 |  |  |  |  |  |
| 11 Positive parenting 3.5y | -0.11 | -0.10 | -0.10 | -0.10 | -0.09 | -0.18 | -0.17 | -0.14 | -0.15 | -0.17 | 1.00 |  |  |  |  |
| 12 Positive parenting 4y | -0.08 | -0.06 | -0.10 | -0.08 | -0.09 | -0.17 | -0.16 | -0.16 | -0.18 | -0.13 | 0.57 | 1.00 |  |  |  |
| 13 Positive parenting 5y | -0.07 | -0.05 | -0.05 | -0.02 | 0.00 | -0.03 | -0.05 | -0.04 | -0.10 | -0.06 | 0.45 | 0.46 | 1.00 |  |  |
| 14 Positive parenting 6y | -0.09 | -0.07 | -0.10 | -0.08 | -0.07 | -0.11 | -0.07 | -0.12 | -0.17 | -0.13 | 0.46 | 0.47 | 0.58 | 1.00 |  |
| 15 Positive parenting 8y | -0.07 | -0.05 | -0.10 | -0.08 | -0.07 | -0.08 | -0.07 | -0.12 | -0.12 | -0.12 | 0.33 | 0.32 | 0.44 | 0.46 | 1.00 |

^a^Data were compiled from the final master file of the Québec Longitudinal Study of Child Development (1998-2021), Gouvernement du Québec, and l’Institut de la Statistique du Québec.

**Table S4.** Factor analysis.

| **Items** | **Factor 1 (Irritability dimension)** | **Factor 2**  **(Hostile/defiant ODD dimension)** |
| --- | --- | --- |
| Reacted in an aggressive manner when contradicted – 3.5y | **0.54** | 0.01 |
| Reacted in an aggressive manner when teased – 3.5y | **0.54** | -0.14 |
| Reacted in an aggressive manner when something was taken away from him/her – 3.5y | **0.50** | 0.03 |
| Reacted in an aggressive manner when contradicted – 4y | **0.50** | 0.07 |
| Reacted in an aggressive manner when teased – 4y | **0.54** | -0.05 |
| Reacted in an aggressive manner when something was taken away from him/her – 4y | **0.51** | 0.03 |
| Reacted in an aggressive manner when contradicted – 5y | **0.62** | 0.02 |
| Reacted in an aggressive manner when teased – 5y | **0.65** | -0.07 |
| Reacted in an aggressive manner when something was taken away from him/her – 5y | **0.57** | 0.05 |
| Reacted in an aggressive manner when contradicted – 6y | **0.61** | 0.03 |
| Reacted in an aggressive manner when teased – 6y | **0.60** | -0.02 |
| Reacted in an aggressive manner when something was taken away from him/her – 6y | **0.57** | 0.01 |
| Had temper tantrums/hot temper – 6y | **0.50** | 0.12 |
| Reacted in an aggressive manner when contradicted – 8y | **0.65** | 0.00 |
| Reacted in an aggressive manner when teased – 8y | **0.61** | -0.02 |
| Reacted in an aggressive manner when something was taken away from him/her – 8y | **0.54** | 0.02 |
| Had temper tantrums/hot temper – 8y | **0.55** | 0.07 |
| Was defiant – 3.5y | 0.25 | **0.31** |
| Didn’t seem to feel guilty after misbehaving – 3.5y | -0.08 | **0.53** |
| Punishment didn’t change his/her behavior – 3.5y | 0.02 | **0.53** |
| Was defiant – 4y | 0.19 | **0.42** |
| Didn’t seem to feel guilty after misbehaving – 4y | -0.03 | **0.55** |
| Punishment didn’t change his/her behavior – 4y | -0.04 | **0.65** |
| Was defiant – 5y | 0.23 | **0.40** |
| Didn’t seem to feel guilty after misbehaving – 5y | -0.05 | **0.60** |
| Punishment didn’t change his/her behavior – 5y | -0.04 | **0.66** |
| Was defiant – 6y | 0.20 | **0.44** |
| Didn’t seem to feel guilty after misbehaving – 6y | 0.02 | **0.51** |
| Punishment didn’t change his/her behavior – 6y | -0.01 | **0.62** |
| Was defiant – 8y | 0.25 | **0.36** |
| Didn’t seem to feel guilty after misbehaving – 8y | 0.08 | **0.44** |
| Punishment didn’t change his/her behavior – 8y | 0.08 | **0.50** |

Abbreviation: ODD, oppositional defiant disorder

**Table S5**. Harsh parenting items at each age.

| **Harsh parenting ages** | **Items** |
| --- | --- |
| 5y, 6y, 8y | How often did you grab firmly or shake the child when he was difficult? |
| 3.5y, 4y, 5y, 6y | How often did you get angry with the child for saying or doing something he was not supposed to? |
| 3.5y, 4y, 5y, 6y, 8y | How often did you hit the child when he was difficult? |
| 3.5y, 4y, 5y, 6y | How often do you get angry when you punish the child? |
| 5y, 6y | How often did you have to discipline him for the same thing? |
| 3.5y, 4y, 5y, 6y, 8y | When the child broke the rules or did things that he was not supposed to, how often did you raise your voice, scold or yell at him? |
| 3.5y, 4y, 5y, 6y, 8y | When the child broke the rules or did things that he was not supposed to, how often did you use physical punishment? |

**Table S6**. Positive parenting items at each age.

| **Positive parenting ages** | **Items** |
| --- | --- |
| 3.5y, 4y, 5y, 6y, 8y | How often did you and the child talk or play with each other,  focusing attention on each other for five minutes or more, just for fun? |
| 3.5y, 4y, 5y, 6y, 8y | How often did you do something special with him that he enjoys? |
| 3.5y, 4y, 5y, 6y, 8y | How often did you play sports activities, hobbies or play games with him? |
| 3.5y, 4y, 5y, 6y | When the child broke the rules or did things that he was not supposed to, how often did you calmly discuss the problem? |
| 3.5y, 4y, 5y, 6y | When the child broke the rules or did things that he was not supposed to, how often did you describe alternative ways of behaving that are acceptable? |
| 5y, 6y, 8y | How often did you play fight with the child just for fun? |
| 5y, 6y, 8y | How often did you say to your child that you were proud of him? |
| 5y, 6y | How often did you help your child doing tasks that were difficult for him? |
| 5y, 6y | How often did you comfort your child when he was sad? |
| 6y | All the times that you talked to the child about behavior, what proportion was praise? |

**Table S7**. Mean scores of irritability and parenting variables for the overall sample and by profile.^a^

|  | **Whole sample** | **Profile 1**  Low irritability,  low harsh parenting,  high positive parenting | **Profile 2**  Moderate irritability, moderate harsh parenting, low positive parenting | **Profile 3**  Moderate irritability, moderate harsh parenting, high positive parenting | **Profile 4**  High irritability, high harsh parenting, low positive parenting |
| --- | --- | --- | --- | --- | --- |
| 1 Irritability 3.5y | 0.72 | 0.47 | 0.70 | 0.81 | 1.06 |
| 2 Irritability 4y | 0.69 | 0.41 | 0.67 | 0.81 | 1.05 |
| 3 Irritability 5y | 0.63 | 0.31 | 0.62 | 0.76 | 1.03 |
| 4 Irritability 6y | 0.69 | 0.37 | 0.65 | 0.82 | 1.14 |
| 5 Irritability 8y | 0.67 | 0.30 | 0.61 | 0.83 | 1.15 |
| 6 Harsh parenting 3.5y | 0.66 | 0.44 | 0.68 | 0.71 | 0.99 |
| 7 Harsh parenting 4y | 0.63 | 0.42 | 0.63 | 0.67 | 0.95 |
| 8 Harsh parenting 5y | 0.54 | 0.35 | 0.55 | 0.59 | 0.82 |
| 9 Harsh parenting 6y | 0.52 | 0.34 | 0.52 | 0.56 | 0.80 |
| 10 Harsh parenting 8y | 0.37 | 0.23 | 0.36 | 0.40 | 0.59 |
| 11 Positive parenting 3.5y | 1.32 | 1.43 | 1.12 | 1.42 | 1.23 |
| 12 Positive parenting 4y | 1.31 | 1.41 | 1.11 | 1.41 | 1.21 |
| 13 Positive parenting 5y | 1.20 | 1.26 | 1.02 | 1.31 | 1.15 |
| 14 Positive parenting 6y | 1.15 | 1.23 | 0.99 | 1.24 | 1.08 |
| 15 Positive parenting 8y | 1.19 | 1.27 | 1.04 | 1.30 | 1.11 |

**Table S8**. Model fit indices.

| **n**  **classes** | **k** | **Loglikelihood** | **AIC** | **BIC** | **SSABIC** | **Entropy** | **Class 1 (%)** | **Class 2 (%)** | **Class 3 (%)** | **Class 4 (%)** | **Class 5 (%)** |
| --- | --- | --- | --- | --- | --- | --- | --- | --- | --- | --- | --- |
| 1 | 21 | -27679.06 | 55400.12 | 55513.39 | 55446.68 | - | 100% | - | - | - | - |
| 2 | 28 | -26088.65 | 52233.29 | 52384.32 | 52295.37 | 0.791 | 53.57% | 46.43% | - | - | - |
| 3 | 35 | -25611.02 | 51292.04 | 51480.83 | 51369.64 | 0.795 | 32.90% | 51.85% | 15.25% | - | - |
| 4 | 42 | -25247.11 | 50578.23 | 50804.77 | 50671.34 | 0.767 | 28.35% | 14.82% | 26.57% | 30.26% | - |
| 5 | 49 | -25087.64 | 50273.29 | 50537.59 | 50381.92 | 0.766 | 11.01% | 23.31% | 23.55% | 12.36% | 29.77% |

**Table S9.** Parameters estimates from the 4-latent class growth model of irritability and parenting.

|  | **Estimate** | **S.E.** | **Estimate/S.E.** | ***p*** |
| --- | --- | --- | --- | --- |
| **Profile 1 (n = 492)** |  |  |  |  |
| Irritability |  |  |  |  |
| Intercept | 0.433 | 0.025 | 17.108 | 0.000 |
| Slope | -0.032 | 0.006 | -5.616 | 0.000 |
| Harsh parenting |  |  |  |  |
| Intercept | 2.214 | 0.071 | 31.272 | 0.000 |
| Slope | -0.234 | 0.013 | -18.686 | 0.000 |
| Positive parenting |  |  |  |  |
| Intercept | 6.965 | 0.103 | 67.572 | 0.000 |
| Slope | -0.238 | 0.018 | -13.361 | 0.000 |
| **Profile 2 (n = 432)** |  |  |  |  |
| Irritability |  |  |  |  |
| Intercept | 0.688 | 0.043 | 16.024 | 0.000 |
| Slope | -0.018 | 0.008 | -2.266 | 0.023 |
| Harsh parenting |  |  |  |  |
| Intercept | 3.328 | 0.120 | 27.839 | 0.000 |
| Slope | -0.338 | 0.016 | -21.662 | 0.000 |
| Positive parenting |  |  |  |  |
| Intercept | 5.544 | 0.094 | 59.196 | 0.000 |
| Slope | -0.145 | 0.018 | -8.042 | 0.000 |
| **Profile 3 (n = 461)** |  |  |  |  |
| Irritability |  |  |  |  |
| Intercept | 0.790 | 0.037 | 21.245 | 0.000 |
| Slope | 0.002 | 0.008 | 0.306 | 0.760 |
| Harsh parenting |  |  |  |  |
| Intercept | 3.509 | 0.116 | 30.351 | 0.000 |
| Slope | -0.335 | 0.017 | -20.118 | 0.000 |
| Positive parenting |  |  |  |  |
| Intercept | 6.952 | 0.067 | 103.707 | 0.000 |
| Slope | -0.195 | 0.020 | -9.731 | 0.000 |
| **Profile 4 (n = 241)** |  |  |  |  |
| Irritability |  |  |  |  |
| Intercept | 1.028 | 0.039 | 26.324 | 0.000 |
| Slope | 0.026 | 0.009 | 2.885 | 0.004 |
| Harsh parenting |  |  |  |  |
| Intercept | 4.888 | 0.133 | 36.775 | 0.000 |
| Slope | -0.421 | 0.034 | -12.256 | 0.000 |
| Positive parenting |  |  |  |  |
| Intercept | 6.062 | 0.158 | 38.484 | 0.000 |
| Slope | -0.184 | 0.024 | -7.675 | 0.000 |

**Table S10.** Descriptive statistics stratified by trajectories of childhood irritability and parenting.^a^

|  | **Profile 1**  Low irritability,  low harsh parenting,  high positive parenting | **Profile 2**  Moderate irritability, moderate harsh parenting, low positive parenting | **Profile 3**  Moderate irritability, moderate harsh parenting, high positive parenting | **Profile 4**  High irritability, high harsh parenting, low positive parenting | ***p*** |
| --- | --- | --- | --- | --- | --- |
| *n* | 492 | 432 | 461 | 241 |  |
| **Child characteristics** |  |  |  |  |  |
| Child sex = girls, *n* (%) | 282 (57.3%) | 222 (51.4%) | 232 (50.3%) | 110 (45.6%) | .018* |
| Low birthweight (<2500), *n* (%) | 14 (2.8%) | 12 (2.8%) | 18 (3.9%) | 6 (2.5%) | .665 |
| Birth order, *n* (%) |  |  |  |  | <.001** |
| 1 | 223 (45.3%) | 141 (32.6%) | 243 (52.7%) | 112 (46.5%) |  |
| 2 | 185 (37.6%) | 205 (47.5%) | 167 (36.2%) | 94 (39.0%) |  |
| 3+ | 84 (17.1%) | 86 (19.9%) | 51 (11.1%) | 35 (14.5%) |  |
| Externalizing problems, mean (*SD*) | 1.68 (0.72) | 2.22 (0.76) | 2.40 (0.76) | 2.83 (0.83) | <.001** |
| Internalizing problems, mean (*SD*) | 1.37 (0.87) | 1.72 (0.87) | 1.88 (0.98) | 2.19 (1.02) | <.001** |
| **Child outcomes** |  |  |  |  |  |
| Irritability, mean (*SD*) | 0.38 (0.28) | 0.67 (0.32) | 0.85 (0.32) | 1.14 (0.37) | <.001** |
| Suicide attempt, mean (*SD*) | 35 (7.1) | 33 (7.6) | 42 (9.1) | 35 (14.5) | .007** |
| **Family characteristics** |  |  |  |  |  |
| Positive parenting, mean (*SD*) | 6.64 (0.68) | 5.28 (0.55) | 6.71 (0.58) | 5.80 (0.73) | <.001** |
| Harsh parenting, mean (*SD*) | 1.82 (0.54) | 2.78 (0.57) | 2.96 (0.56) | 4.23 (0.71) | <.001** |
| Socioeconomic status (SES), mean (*SD*) | 0.16 (0.95) | 0.05 (0.92) | -0.04 (0.96) | -0.20 (0.96) | <.001** |
| Family structure, *n* (%) |  |  |  |  | .010** |
| Intact | 400 (81.5%) | 366 (84.9%) | 372 (81.2%) | 186 (77.5%) |  |
| Single | 36 (7.3%) | 19 (4.4%) | 29 (6.3%) | 30 (12.5%) |  |
| Blended | 55 (11.2%) | 46 (10.7%) | 57 (12.4%) | 24 (10.0%) |  |
| Maternal depression, mean (*SD*) | 1.10 (1.14) | 1.47 (1.31) | 1.33 (1.27) | 1.85 (1.60) | <.001** |
| Paternal depression, mean (*SD*) | 0.84 (0.87) | 1.08 (0.98) | 1.00 (0.96) | 1.12 (0.96) | .001** |
| Maternal age, mean (*SD*) | 30.04 (5.12) | 29.43 (4.87) | 29.20 (5.32) | 28.38 (5.29) | .001** |
| Paternal age, mean (*SD*) | 32.61 (5.46) | 32.11 (5.20) | 32.29 (5.87) | 31.73 (5.49) | .249 |

*Note.* ^a^Data were compiled from the final master file of the Québec Longitudinal Study of Child Development (1998-2021), Gouvernement du Québec, and l’Institut de la Statistique du Québec. Externalizing, internalizing problems, and SES, measured from 1.5 to 8y; irritability, positive, and harsh parenting measured from 3.5y to 8y. Family structure, maternal age and depression, and paternal age and depression, measured at 5 months.

**p* < .05. ***p* <.01.

**Table S11**. Descriptive statistics of suicide attempt at each age.^a^

| **Total suicide attempts (past-year assessments)** | ***N* = 122** |
| --- | --- |
|  |  |
| **Age at first suicide attempt** | ***n (%)*** |
| 13y | 30 (24.6) |
| 15y | 35 (28.7) |
| 17y | 21 (17.2) |
| 20y | 22 (18.0) |
| 23y | 14 (11.5) |
|  |  |
| **Frequency of suicide attempt** | ***n (%)*** |
| Single suicide attempt | 99 (81.1) |
| Repeated suicide attempts | 23 (18.9) |

*Note*. ^a^Suicide attempts reported at each age, excluding additional lifetime hospitalization questions asked at ages 20 and 23.

**Table S12**. Associations of childhood profiles of irritability and parenting at 3.5 to 8 years of age with youth suicide attempt, non-imputed data.^a^

|  |  | **Outcome, OR (95% CI)** | | | |
| --- | --- | --- | --- | --- | --- |
|  | **Suicide Attempt** | |  |  |  |
| **Profile** | **Unadjusted** | | **Adjusted^b^** | **Adjusted^c^** | **Adjusted^d^** |
| Low irritability, low harsh parenting, and high positive parenting | 1 [Reference] | | 1 [Reference] | 1 [Reference] | 1 [Reference] |
| Moderate irritability, moderate harsh parenting, and low positive parenting | 1.08 (0.66-1.77) | | 1.08 (0.65-1.77) | 1.05 (0.63-1.74) | 0.94 (0.56-1.58) |
| Moderate irritability, moderate harsh parenting, and high positive parenting | 1.31 (0.82-2.09) | | 1.28 (0.8-2.05) | 1.26 (0.78-2.03) | 1.08 (0.66-1.8) |
| High irritability, high harsh parenting, and low positive parenting | 2.22 (1.35-3.65) | | 2.16 (1.3-3.59) | 2.14 (1.27-3.59) | 1.68 (0.95-2.99) |

Abbreviation: OR, odds ratio.

^a^Data were compiled from the final master file of the Québec Longitudinal Study of Child Development (1998-2021), Gouvernement du Québec, and l’Institut de la Statistique du Québec.

^b^Adjusted for child sex and socioeconomic status (SES).

^c^Adjusted for child sex, SES, family structure, maternal age, and maternal depression.

^d^Adjusted for child sex, SES, family structure, maternal age, maternal depression, child internalizing, and externalizing problems.

**Table S13**. Associations of childhood profiles of irritability and parenting at 3.5 to 8 years of age with youth suicide attempt based on different reference categories, non-imputed data.^a^

|  |  | **Outcome, OR (95% CI)** | | | |
| --- | --- | --- | --- | --- | --- |
|  | **Suicide Attempt** | |  |  |  |
| **Profile** | **Unadjusted** | | **Adjusted^b^** | **Adjusted^c^** | **Adjusted^d^** |
| Low irritability, low harsh parenting, and high positive parenting | 0.93 (0.56-1.52) | | 0.93 (0.56-1.53) | 0.95 (0.57-1.58) | 1.06 (0.63-1.79) |
| Moderate irritability, moderate harsh parenting, and low positive parenting | 1 [Reference] | | 1 [Reference] | 1 [Reference] | 1 [Reference] |
| Moderate irritability, moderate harsh parenting, and high positive parenting | 1.21 (0.75-1.95) | | 1.19 (0.73-1.92) | 1.2 (0.74-1.95) | 1.15 (0.71-1.88) |
| High irritability, high harsh parenting, and low positive parenting | 2.05 (1.24-3.4) | | 2.01 (1.2-3.35) | 2.03 (1.21-3.42) | 1.79 (1.05-3.06) |
| Low irritability, low harsh parenting, and high positive parenting | 0.76 (0.48-1.22) | | 0.78 (0.49-1.26) | 0.79 (0.49-1.28) | 0.92 (0.56-1.53) |
| Moderate irritability, moderate harsh parenting, and low positive parenting | 0.83 (0.51-1.33) | | 0.84 (0.52-1.36) | 0.83 (0.51-1.36) | 0.87 (0.53-1.41) |
| Moderate irritability, moderate harsh parenting, and high positive parenting | 1 [Reference] | | 1 [Reference] | 1 [Reference] | 1 [Reference] |
| High irritability, high harsh parenting, and low positive parenting | 1.69 (1.05-2.74) | | 1.69 (1.04-2.75) | 1.69 (1.03-2.78) | 1.55 (0.94-2.56) |

Abbreviation: OR, odds ratio.

^a^Data were compiled from the final master file of the Québec Longitudinal Study of Child Development (1998-2021), Gouvernement du Québec, and l’Institut de la Statistique du Québec.

^b^Adjusted for child sex and socioeconomic status (SES).

^c^Adjusted for child sex, SES, family structure, maternal age, and maternal depression.

^d^Adjusted for child sex, SES, family structure, maternal age, maternal depression, child internalizing, and externalizing problems.

**Table S14**. Associations of childhood profiles of irritability and parenting at 3.5 to 8 years of age with youth suicide attempt based on different reference categories, imputed data.^a^

|  |  | **Models, OR (95% CI)** | | | |
| --- | --- | --- | --- | --- | --- |
| **Profile** | **Unadjusted** | | **Adjusted^b^** | **Adjusted^c^** | **Adjusted^d^** |
| Low irritability, low harsh parenting, and high positive parenting | 0.87 (0.53-1.43) | | 0.89 (0.54-1.47) | 0.89 (0.54-1.47) | 0.99 (0.59-1.66) |
| Moderate irritability, moderate harsh parenting, and low positive parenting | 1 [Reference] | | 1 [Reference] | 1 [Reference] | 1 [Reference] |
| Moderate irritability, moderate harsh parenting, and high positive parenting | 1.27 (0.80-2.02) | | 1.26 (0.79-2.00) | 1.25 (0.79-1.99) | 1.20 (0.75-1.91) |
| High irritability, high harsh parenting, and low positive parenting | 2.19 (1.35-3.56) | | 2.10 (1.29-3.44) | 2.03 (1.24-3.33) | 1.78 (1.07-2.97) |
| Low irritability, low harsh parenting, and high positive parenting | 0.69 (0.43-1.09) | | 0.71 (0.44-1.13) | 0.71 (0.44-1.14) | 0.83 (0.50-1.35) |
| Moderate irritability, moderate harsh parenting, and low positive parenting | 0.79 (0.50-1.24) | | 0.79 (0.50-1.26) | 0.80 (0.50-1.27) | 0.83 (0.52-1.33) |
| Moderate irritability, moderate harsh parenting, and high positive parenting | 1 [Reference] | | 1 [Reference] | 1 [Reference] | 1 [Reference] |
| High irritability, high harsh parenting, and low positive parenting | 1.72 (1.10-2.71) | | 1.67 (1.06-2.64) | 1.62 (1.02-2.58) | 1.49 (0.93-2.38) |

Abbreviation: OR, odds ratio.

^a^Data were compiled from the final master file of the Québec Longitudinal Study of Child Development (1998-2021), Gouvernement du Québec, and l’Institut de la Statistique du Québec.

^b^Adjusted for child sex and socioeconomic status (SES).

^c^Adjusted for child sex, SES, family structure, maternal age, and maternal depression.

^d^Adjusted for child sex, SES, family structure, maternal age, maternal depression, child internalizing, and externalizing problems.


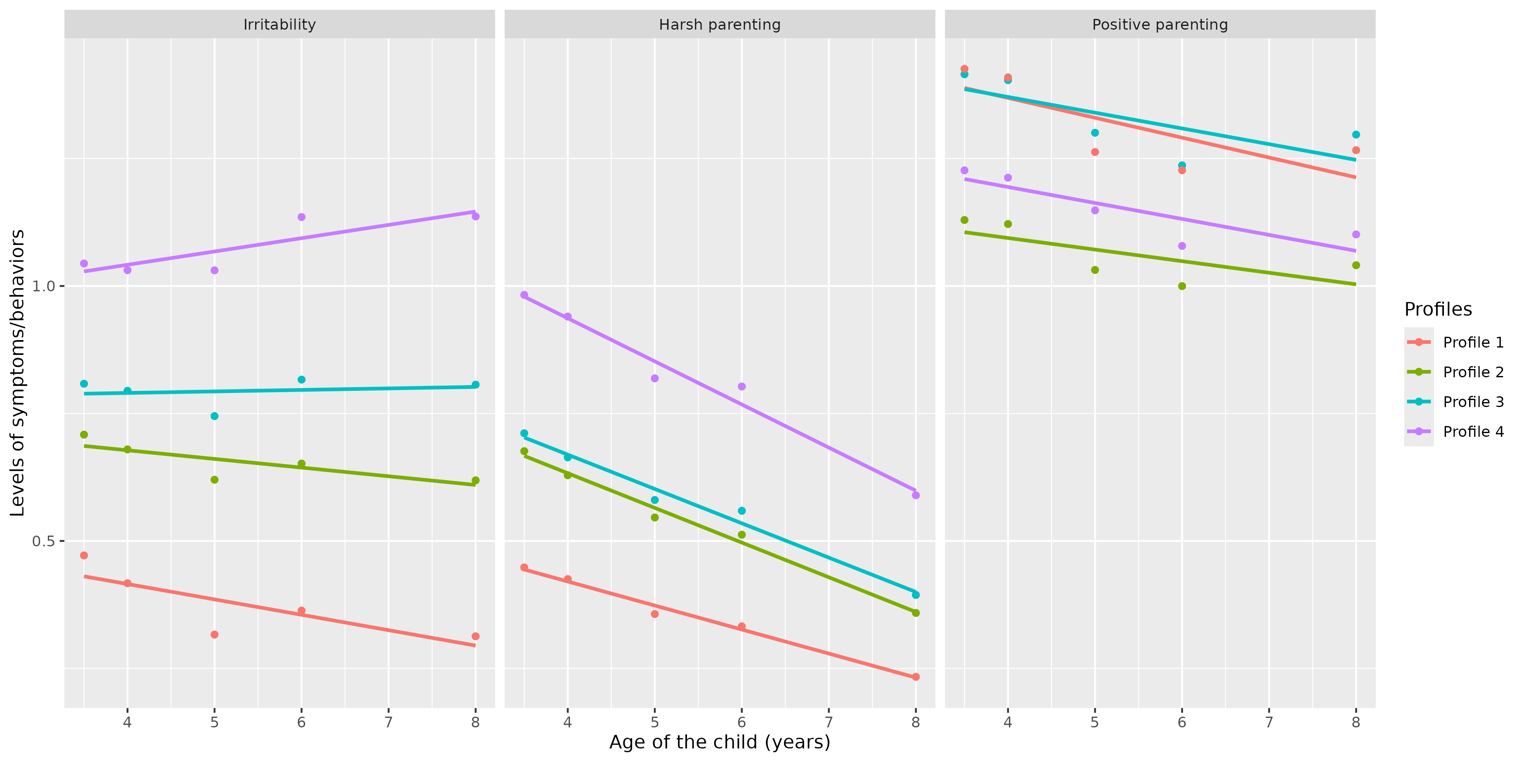


**Figure S1.** Profiles of irritability, harsh parenting, and positive parenting (alternative representation). Data were compiled from the final master file of the Québec Longitudinal Study of Child Development (1998-2021), Gouvernement du Québec, and l’Institut de la Statistique du Québec.
